# Systemic inflammation enhances stimulant-induced striatal dopamine elevation

**DOI:** 10.1038/tp.2017.18

**Published:** 2017-03-28

**Authors:** J R Petrulli, B Kalish, N B Nabulsi, Y Huang, J Hannestad, E D Morris

**Affiliations:** 1 Department of Biomedical Engineering, Yale University, New Haven, CT, USA; 2 Department of Radiology and Biomedical Imaging, Yale University, New Haven, CT, USA; 3 Denali Therapeutics Inc., South San Francisco, CA, USA; 4 Department of Psychiatry, Yale University, New Haven, CT, USA

## Abstract

Changes in the mesolimbic dopamine (DA) system are implicated in a range of neuropsychiatric conditions including addiction, depression and schizophrenia. Dysfunction of the neuroimmune system is often comorbid with such conditions and affects similar areas of the brain. The goal of this study was to use positron emission tomography with the dopamine D_2_ antagonist tracer, ^11^C-raclopride, to explore the effect of acute immune activation on striatal DA levels. DA transmission was modulated by an oral methylphenidate (MP) challenge in order to reliably elicit DA elevation. Elevation in DA concentration due to MP was estimated via change in ^11^C-raclopride binding potential from the baseline scan. Prior to the post-MP scan, subjects were pre-treated with either the immune activator lipopolysaccharide (LPS) or placebo (PBO) in a cross-over design. Immune activation was confirmed by measuring tumor necrosis factor alpha (TNFα), interleukin (IL)-6 and IL-8 concentration in plasma. Eight healthy subjects were scanned four times each to determine the MP-induced DA elevation under both LPS and PBO pre-treatment conditions. MP-induced DA elevation in the striatum was significantly greater (*P*<0.01) after LPS pre-treatment compared to PBO pre-treatment. Seven of eight subjects responded similarly. This effect was observed in the caudate and putamen (*P*<0.02), but was not present in ventral striatum. DA elevation induced by MP was significantly greater when subjects were pre-treated with LPS compared to PBO. The amplification of stimulant-induced DA signaling in the presence of systemic inflammation may have important implications for our understanding of addiction and other diseases of DA dysfunction.

## Introduction

Interaction between the immune and nervous systems in health and disease is an area of growing research focus. The immune system plays a key role in classic neuroimmune diseases such as multiple sclerosis;^1^ however, genetic, imaging, postmortem and animal data also suggest that immune–brain interactions influence the pathophysiology of a broad range of neurological and psychiatric diseases, including neurodegenerative diseases,^2^ epilepsy,^3^ addiction,^4, 5^ depression,^6, 7^ schizophrenia^8, 9^ and autism.^10^ The exact role of immune–brain interactions in these diseases is not fully understood; however, it is well established that experimental manipulation of systemic inflammation produces effects on the human brain. Functional magnetic resonance imaging (fMRI) studies involving induced neuroinflammation have demonstrated increased neural activity in areas implicated in social pain^11^ and a blunted behavioral response to reward.^12, 13^ Using the same methods to induce neuroinflammation, Hannestad *et al.*^14^ observed an increase in glucose metabolism in the insula and a decrease of glucose metabolism in the cingulate using ^18^F-flurodeoxyglucose (FDG) positron emission tomography (PET). Systemic inflammation is known to produce behavioral effects such as change in sleep and appetite, fatigue, anhedonia and depression that are consistent with the roles of these brain regions.^12, 15, 16^ Elevated plasma concentration of inflammatory mediators is associated with these symptoms as well.^17, 18, 19^ Depressed patients with higher levels of inflammatory markers have been found to have less functional connectivity in reward areas such as the ventral striatum and ventromedial prefrontal cortex.^20^ It has also been shown that systemic inflammation can cause widespread activation of microglia in the human brain.^21^ The regions affected by experimental systemic inflammation in imaging studies overlap substantially with regions that are involved in neurologic (basal ganglia, insula and temporal cortex) or psychiatric disorders (cingulate, medial frontal cortex and superior frontal cortex),^22^ and regions that show reduced gray matter in psychiatric diseases (anterior insula/dorsal cingulate network).^23^

Immune activation can be reliably elicited with lipopolysaccharide (LPS), an agonist of the toll-like receptor 4 (TLR4). LPS-induced activation of TLR4 causes systemic inflammation,^14^ depression-like behavior,^15, 16^ activation of microglia^21^ and changes in neuronal activity.^12^ In humans, systemic inflammation has an effect on the response of areas involved in reward (ventral striatum and substantia nigra).^12, 13^ Although the effects of systemic inflammation on behavior, symptoms and gross measures of brain function (for example, fMRI and FDG PET) have been characterized, much less is known about the molecular events that mediate these effects. One potential mediator of the effects of systemic inflammation on behavior is dopamine (DA). fMRI and ^18^F-FDG do not have the specificity to definitively determine DA involvement.

In rodents, acute systemic immune activation with LPS produced increases in DA.^24^ Similarly, in nonhuman primates, interferon-alpha (IFNα) administration caused an increase in stimulated DA release after 2 weeks, but a reversal of this effect was found after 4 weeks.^25^ Levodopa, a DA precursor, was found to restore deficits in DA elevation caused by chronic inflammation.^26^ These data suggest that acute or short-term increases in systemic inflammation lead to an increase in DA, while chronic inflammation has the opposite effect.

PET with ^11^C-raclopride, a DA D_2_ receptor antagonist, has been used reliably to detect elevation of striatal DA after administration of methylphenidate (MP), a DA reuptake inhibitor.^27, 28, 29, 30, 31^ Similarly, ^11^C-raclopride has also been used to characterize the mesolimbic DA system in disorders where DA dysfunction is implicated: smoking dependence,^32^ addiction,^33^ depression,^34^ schizophrenia^35^ and attention-deficit/hyperactivity disorder.^36^ In the present study, we examined the effects of LPS administration on MP-induced DA elevation. To our knowledge, this is the first study in humans to measure changes in DA after experimental activation of the immune system.

## Materials and methods

### Design overview

**Figure 1:**
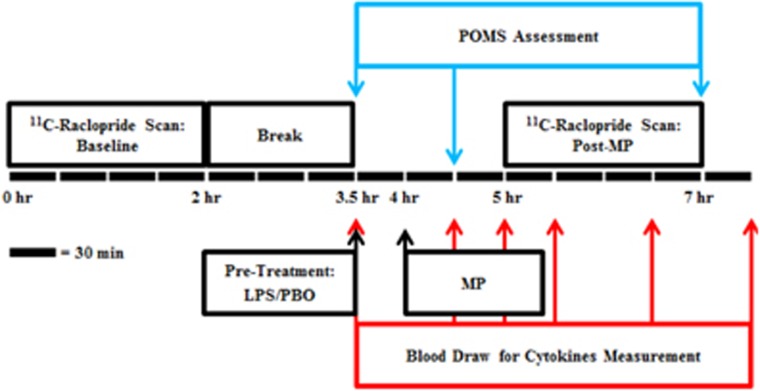
Schematic depiction of a session containing two ^11^C-raclopride PET scans: baseline and post MP. Each subject underwent two of these sessions with different pre-treatments: LPS or placebo. The order of the sessions was randomized. Blood sampling and POMS questions were performed periodically (denoted by arrows) from the start of pre-treatment until the end of the post-MP scan. LPS, lipopolysaccharide; MP, methylphenidate; PET, positron emission tomography; PBO, placebo; POMS, Profile of Mood States.

This study was approved by Yale University's Human Investigation Committee, Radioactive Drug Research Committee, and Radiation Safety Committee. Eight healthy subjects (four women and four men, mean age 31.3±11.2 years) without significant medical issues, current or recent smoking or other nicotine use, and personal or family history of psychiatric disorders (including substance misuse) were recruited for this study. Eligibility was confirmed by a detailed clinical interview, medical history, a physical exam, screening labs, urine cotinine and various assessments, including the Beck Anxiety Inventory, Hamilton Depression Rating Scale and Cognitive Emotion Regulation Questionnaire. Informed consent was obtained prior to the performance of any study procedures. Each subject underwent a structural MRI scan on a Trio 3T MR scanner (Siemen's/CTI, Knoxville, TN, USA). Subjects then participated in two PET scan sessions on two separate days. Each scan session consisted of a pair of PET scans: a baseline scan and a post-MP scan. Sessions began with a baseline 120 min ^11^C-raclopride scan. Subjects then rested for ~90–120 min. Following the break, a pre-treatment of either LPS (0.8 ng kg^−^^1^) or saline placebo (PBO) was administered intravenously. After 30 min, subjects received oral MP (40 mg). The post-MP ^11^C-raclopride scan was then performed 60 min after MP administration (90 min after LPS/PBO pre-treatment) for 120 min (Figure 1). Subjects returned at least 1 week later for a second scan session that followed the same sequence (baseline scan, break, pre-treatment, MP administration, post-MP scan) but with the opposite pre-treatment condition (LPS/PBO). The order of pre-treatment was randomized and both subjects and experimenters were blinded to the condition until after data processing was completed. The dose of LPS was selected based on previous studies that produced changes in brain activity^12, 14^ and induced fatigue.^15, 16^ The dose of MP was determined using previous studies that reported a robust change in raclopride signal.^29, 30^

Two additional subjects participated in a single scan session with only LPS pre-treatment without MP to assess the effect of LPS alone on DA levels. This session consisted of a baseline scan, break, pre-treatment with LPS and a post-LPS scan.

### PET scans

Subjects were permitted a light breakfast (without caffeine) prior to scanning. An intravenous catheter was placed for ^11^C-raclopride injection and periodic blood sampling. Blood pressure, heart rate and temperature were monitored by medical staff to ensure subject well-being. ^11^C-raclopride was synthesized using methods previously described in Langer *et al.*^37^ The radiotracer was injected intravenously as a bolus containing a mean activity of 17.4±3.4 mCi and a mean mass of 0.92±0.35 μg. Neither injected activity nor mass was significantly different between baseline, MP+PBO, and MP+LPS scans (*P*=0.83 and *P*=0.68, respectively) as characterized by a single factor analysis of variance. Subjects were placed on the scanner bed with their head centered at the field of view; a 9 min transmission scan (^68^Ge rods, 511 keV source) was obtained for attenuation correction. PET scans were acquired for 120 min on an ECAT HR+ PET scanner (Siemen's/CTI) that has a spatial resolution of 4.3–8.3 mm FWHM.^38^

### Blood and behavior analyses

Samples of venous blood (10 ml) were drawn at 30, 90 and 150 min post-MP administration to assess MP concentration. Additional 10 ml venous blood samples were drawn at 0, 60, 90, 120, 180 and 240 min post LPS injection (Figure 1) to measure concentrations of the cytokines: tumor necrosis factor alpha (TNFα), interleukin 6 (IL-6) and IL-8. Blood samples were centrifuged for 10 min and the plasma stored at −80 °C. Plasma samples were assayed for MP (Nathan Kline Institute, Orangeburg, NY, USA) and cytokines (Yale Cancer Center Immune Monitoring Lab, New Haven, CT, USA).

The Profile of Mood States questionnaire (POMS^39^) was administered to all subjects at 0, 60 and 210 min after pre-treatment with LPS or PBO. POMS is a Likert scale containing various mood states that are each rated from 0 to 4. For this analysis, we examined the ‘fatigued' mood state because, based on previous studies by our group, this symptom was reliably induced by LPS alone.^15, 21^

### Image analysis

PET emission data were collected in 3D. Two-dimensional sinograms were created with Fourier rebinning. Data were binned into time frames of 6 × 0.5, 3 × 1, 2 × 2 and 22 × 5 min. Data were corrected for attenuation, scatter, dead time, detector sensitivity and randoms. Images were reconstructed with ordered subset expectation maximization using 4 iterations and 16 subsets (in-house software) at a voxel size of 2.1 mm × 2.1 mm × 2.4 mm and image volume of 128 × 128 × 63 voxels. PET images were co-registered to the subject's respective MR scan (FMRIB's Software Library^40^) and then non-linearly registered to a MR template in a common space (Bioimage Suite^41, 42^). ROIs were extracted using automated anatomic labeling^43^ (AAL) in order to generate regional time activity curves. The whole striatum, caudate, and putamen ROIs were extracted using the AAL atlas. The ventral striatum ROI was based on Martinez *et al.*^44^ Time activity curves were fitted with the simplified reference tissue model^45^ using the cerebellum as a reference region in order to calculate non-displaceable binding potential (BP_ND_; abbreviated here as BP). Test–retest reliability of BP was assessed by comparing BP values for the caudate, putamen and ventral striatum from each of the two baseline scans for each subject using interclass correlation and a Wilcoxon signed-rank test. Change in BP was calculated between baseline and post MP for both LPS and PBO pre-treatment conditions:


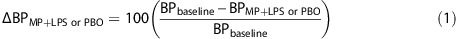


ΔBP was compared between conditions for each ROI using the Student's paired *t*-test (two-tailed) to produce *P-*values (*P*<0.05 considered significant, uncorrected). BP was also calculated at the voxel level for each subject and used to construct mean, voxel-wise ΔBP images for each condition. *T*-tests were applied voxel by voxel to detect differences in ΔBP by condition. In order to examine potential relationships between MP concentration, cytokine concentration, ΔBP and change in POMS scores, the Pearson correlation coefficients (and associated *P-*values) were used.

## Results

### Blood and behavior analysis

**Figure 2:**
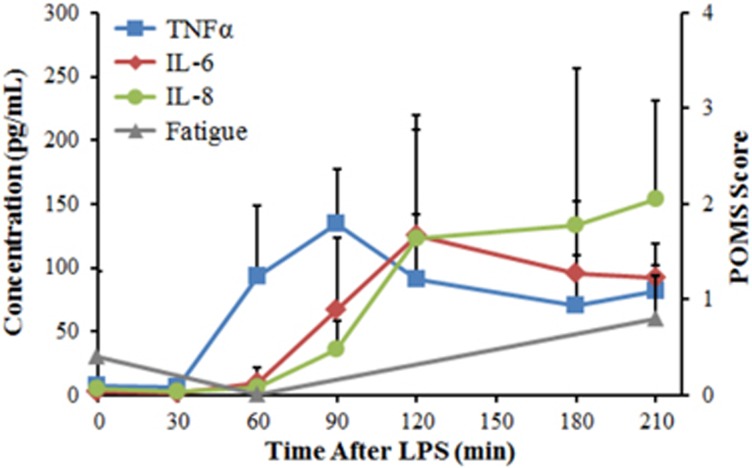
Mean cytokine concentration of TNFα (▪), IL-6 (♦) and IL-8 (•) in MP+LPS scans (*n*=8). The corresponding mean POMS score of fatigue is plotted on a secondary axis (▴). Error bars are shown as the standard deviation between subjects. IL, interleukin; LPS, lipopolysaccharide; MP, methylphenidate; POMS, Profile of Mood States; TNFα, tumor necrosis factor alpha.

The mean concentration of MP in plasma during the scan was not different between the MP+LPS (12.7±7.4 ng ml^−1^) and MP+PBO (11.7±5.2 ng ml^−1^) conditions (*P*>0.6). MP concentration did not correlate with ΔBP in either the MP+PBO (*r*=0.5; *P*>0.2) or MP+LPS condition (*r*=0.4; *P*>0.3). Mean cytokine concentrations for TNFα, IL-6, and IL-8 (Figure 2) were consistent with previous LPS studies by our group^15, 16^ and others.^12^ The measured concentrations of cytokines were not correlated with ΔBP_MP+LPS_ (*r*<0.5; *P*>0.2). POMS fatigue score did not change significantly from baseline in MP+LPS or MP+PBO conditions at 60 and 210 min post LPS or PBO administration (*P*>0.2). Change from baseline fatigue scores taken at the 60 or 210 min time points did not correlate with ΔBP in either the MP+PBO or MP+LPS condition (*r*<0.5; *P*>0.2). Concentration of MP did not correlate with cytokine concentrations or POMS scores (*r*<0.4; *P*>0.3).

### Image analysis

**Figure 3:**
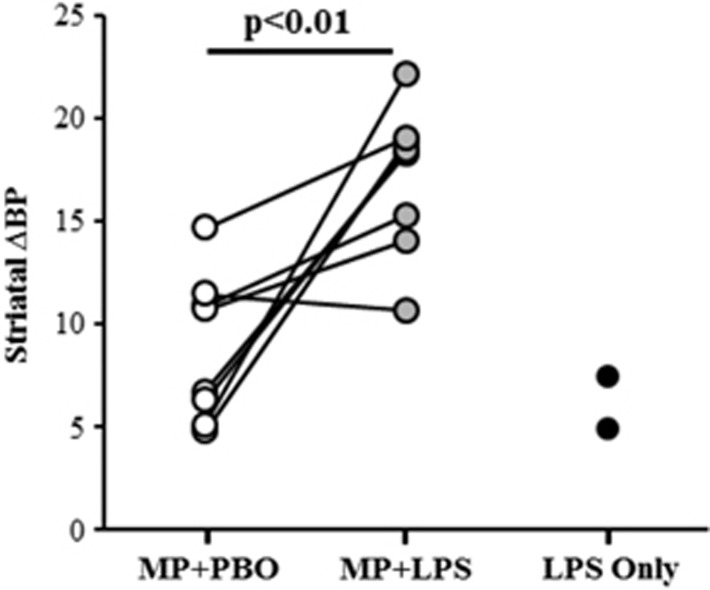
ΔBP values in striatum from each subject in each condition. Seven of eight subjects exhibited greater ΔBP after MP+LPS administration (gray) versus MP+PBO (white; *P*<0.01). Line segments were added to indicate the same subject in different conditions. Preliminary results (*n*=2) of ΔBP after LPS alone are plotted in black in the right-most column. These subjects did not receive any other scan condition. BP, binding potential; LPS, lipopolysaccharide; MP, methylphenidate; PBO, placebo.

**Figure 4:**
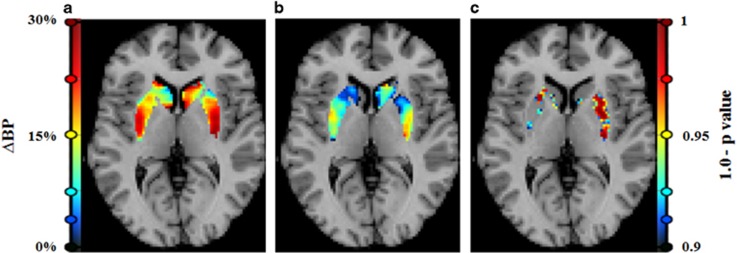
Images created from SRTM modeling at the voxel level. Analyses were performed on images aligned to a common space and averaged: (**a**) mean ΔBP_MP+LPS_ image, (**b**) mean ΔBP_MP+PBO_ image and (**c**) significance image created by paired *t*-testing ΔBP between drug conditions at each voxel. Voxel intensity is calculated as (1.0−*P-*value). Voxel values are only displayed where (1.0−*P-*value)>0.90. In this two-tailed *t*-test, no voxels were found to be significant for ΔBP_MP+LPS_<ΔBP_MP+PBO_. The sparsity of significant differences on the left side of the brain is likely due to greater observed variability in the MP+PBO condition in left ROIs (see Table 1 for ROI level values). BP, binding potential; LPS, lipopolysaccharide; MP, methylphenidate; PBO, placebo; SRTM, simplified reference tissue model.

Baseline BP was not different between pre-treatment conditions in the striatum (*P*=0.82) or striatal sub-regions (ICC=0.95, Wilcoxon *P*=1.0). Intra-subject percent variability in baseline BP was 5.2%. Whole striatum ΔBP was greater in the MP+LPS condition (ΔBP_MP+LPS_=17.1±3.6) compared to the MP+PBO condition (ΔBP_MP+PBO_=8.8±3.6). This finding was consistent: seven of eight subjects exhibited greater ΔBP_MP+LPS_ compared to ΔBP_MP+PBO_ in whole striatum (*P*=0.007), shown in Figure 3. Striatal results were robust enough to survive Bonferroni correction for 6 independent measurements (left/right caudate, putamen, and ventral striatum; *P*<0.05/6). Analysis by sub-region showed that mean ΔBP_MP+LPS_ was greater than mean ΔBP_MP+PBO_ in both the caudate (*P*<0.02) and the putamen (*P*<0.02), but not in the ventral striatum (*P*>0.8; see sub-region results in Table 1). Voxel-wise ΔBP images were consistent with ROI-level findings: significant differences were only found in voxels for which ΔBP_MP+LPS_>ΔBP_MP+PBO_ (Figure 4). The percentage of voxels found to be significantly different between pre-treatment conditions was 34.3% in putamen, 23.5% in caudate and 5.5% in ventral striatum.

In the two subjects who did not receive MP, the mean ΔBP_LPS_=6.1±1.8 in whole striatum (Figure 3 and Table 1). Cytokine levels for these two control subjects were consistent with the eight subjects who received the MP+LPS condition. POMS fatigue scores in the LPS alone condition increased by 1 in one subject and by 2 in the other subject.

## Discussion

In the striatum, BP at baseline was significantly greater than BP post MP in both the MP+PBO condition (*P*<0.0005) and in MP+LPS condition (*P*<0.0005; Supplementary Figure 1). ΔBP due to MP+PBO was comparable to that found in other studies using similar MP doses and routes of administration.^29, 30^ The effects we observed (either due to MP+PBO or MP+LPS) were consistently greater than the calculated test–retest variability in baseline BP in our data (5.2%) as well as in other studies.^27, 31^

Post-MP scans were initiated 90 min after LPS administration and image acquisition continued for 120 min, a time period during which the peak effects of LPS on blood cytokine levels,^12, 15, 16^ neuronal function (glucose metabolism in ^18^F-FDG PET^14^ or BOLD signal in fMRI^11, 46^) and behavioral effects (fatigue and other sickness symptoms) occur.^12, 15, 16, 21, 47^ Plasma analyses confirmed that cytokines were elevated as anticipated post LPS administration in a manner consistent with prior studies (Figure 2).^12, 15, 16^

Four subjects underwent the LPS pre-treatment session before the PBO pre-treatment session and four subjects received pre-treatment sessions in the opposite order. No significant difference in ΔBP_MP+LPS_ was observed between subjects whether LPS was given first or PBO was given first. The same result was found in ΔBP_MP+PBO_.

In previous studies, LPS administration produced a clear effect on fatigue;^15, 16^ however, in this study we did not observe fatigue when LPS was co-administered with MP. We speculate that MP partially counteracts LPS-induced behavioral effects through a central (brain) mechanism, rather than a peripheral mechanism, as MP had no effect on circulating levels of cytokines (*r*<0.4; *P*>0.3). This decoupling of LPS-induced behavioral effects (fatigue and other sickness symptoms) and immune effects (blood cytokine levels) in the presence of MP is a novel result which indicates that blood cytokine concentrations may not be reliable indicators of the effects of systemic inflammation on brain-related symptoms. The potential effect of elevated cytokines in blood on the brain will depend on the susceptibility of the brain to these influences. In this study, it appears that MP had an effect on the brain that prevented the typical feeling of fatigue induced by LPS. Since both LPS alone and MP alone produce similar increases in DA, our data suggest that striatal DA levels do not have a direct relationship with fatigue. It is possible that DA in other brain regions is implicated in fatigue, or that other effects of MP counteracted fatigue (for example, the inhibition of norepinephrine uptake).^48^

Prior studies have suggested a cross sensitization between stressors and DA changes.^49, 50^ But, there appear to be differences between the effects of short- and long-term neuroimmune activation. In rodents, LPS induced an increase in DA concentration, an effect which peaked 2 h after LPS administration.^24^ Mice acutely pre-treated with LPS and cocaine displayed greater locomotion in a behavioral task compared to pre-treatment with cocaine alone.^51^ In nonhuman primates, 2 weeks of IFNα (a cytokine involved in inflammatory signaling) caused an increase in amphetamine-stimulated DA release over amphetamine alone.^25^ Our findings are consistent with prior studies, namely, stimulated DA elevation was enhanced with an acute dose of LPS compared to placebo. Studies of chronic inflammation have not found a synergistic effect of inflammation on DA activity. Capuron *et al.*^52^ found reduced ^18^F-DOPA uptake in humans after chronic IFNα administration. Chronic treatment with IFNα was also observed to reduce striatal dopamine release in primates as measured by microdialysis.^26^ In fact, the same monkeys studied by Felger *et al.*^25^ that experienced cytokine-enhanced DA release after 2 weeks showed a reversal of this effect when IFNα administration was extended to 4 weeks.

Imaging studies in humans have demonstrated a link between neuroinflammation and activity in the striatum. However, there appear to be differences in the effects on the ventral versus dorsal striatum. Using fMRI, Felger *et al.*^20^ showed a decrease in ventral striatal connectivity (during resting state) in depressed individuals with neuroinflammation. In non-depressed populations, ventral striatal BOLD activity in response to reward was blunted by both an acute dose of LPS^12, 13^ and chronic IFNα administration.^52^ The findings of these fMRI studies were localized to ventral striatum and indicate a reduction in reward reactivity during immune activation. In contrast, the present study found a synergistic effect of immune activity on DA elevation only in the dorsal striatum (caudate and putamen). DA in the dorsal (as opposed to ventral) striatum has been linked to response inhibition, another important behavioral characteristic of substance abuse.^53, 54, 55^ Low DA receptor availability in the dorsal striatum (as measured by PET imaging in humans) has been associated with impaired response inhibition.^56, 57, 58, 59^ The results of the present study are consistent with this body of work and represent a novel contribution to it, namely, that DA activity in response to a stressor is enhanced in the dorsal striatum.

The interaction between an immune response and DA signaling may carry unappreciated risks. If in the presence of inflammation, a dopaminergic medication or other stimulus with addiction potential produces higher DA elevation in the dorsal striatum than without, then it is reasonable to expect that the addiction liability of the stimulus may be increased through impaired response inhibition. Consider an individual experimenting with illicit stimulants or an adolescent prescribed Ritalin (MP), either of whom are experiencing short-term neuroinflammation. Both could be at increased risk for addiction due to reduced inhibitory control caused by a supraphysiologic DA elevation. We believe our findings call for further investigation of patient populations who may be suffering from neuroinflammation and are using or abusing stimulants.

## Conclusion

MP-induced DA elevation in the striatum was increased in the presence of LPS versus placebo (seven of eight subjects, *P*<0.01). Co-administration of MP and LPS elevated subjects' inflammatory cytokine concentrations but did not elicit negative behavioral effects typically observed during systemic inflammation. The synergistic effect between immune activation and DA stimulants could have important implications in the treatment of neuroimmune compromised and DA dysfunctional populations.

## Figures and Tables

**Table 1:** Mean (standard deviation) change in binding potential from baseline in MP+LPS, MP+PBO, LPS pre-treatment conditions

*ROI*	*Volume (mL)*	*Mean ΔBP (*n=*8)*		P-v*alue*	*Mean ΔBP (*n=*2)*
		*MP+LPS*	*MP+PBO*		*LPS*
Striatum	42.6	17.1 (3.6)	8.8 (3.6)	0.007	6.1 (1.8)

*Putamen*	21.9	17.9 (5.0)	9.5 (4.5)	0.013	4.5 (3.2)
Left	10.7	18.0 (5.4)	9.7 (7.5)	0.071	4.7 (0.1)
Right	11.3	17.7 (5.6)	9.1 (4.9)	0.001	4.6 (5.6)

*Caudate*	20.7	16.1 (5.4)	7.7 (3.7)	0.020	8.6 (0.2)
Left	10.2	13.9 (7.8)	4.8 (9.2)	0.099	9.6 (3.3)
Right	10.5	18.5 (5.3)	11.0 (11.6)	0.058	7.3 (3.7)

*Ventral striatum*	2.9	12.7 (8.1)	11.4 (9.3)	0.809	1.7 (5.4)
Left	1.4	16.6 (7.1)	11.5 (11.4)	0.409	3.9 (7.5)
Right	1.5	7.3 (13.8)	10.8 (13.5)	0.574	−1.3 (2.9)

Abbreviations: ΔBP, change in binding potential; LPS, lipopolysaccharide; MP, methylphenidate; PBO, placebo.
