# Supplementary figures and images for: Systemic inflammation enhances stimulant-induced striatal dopamine elevation
Source: Transl Psychiatry. 2017 Mar 28;7(3):e1076–. doi: 10.1038/tp.2017.18 (PMC5404612; doi:10.1038/tp.2017.18)

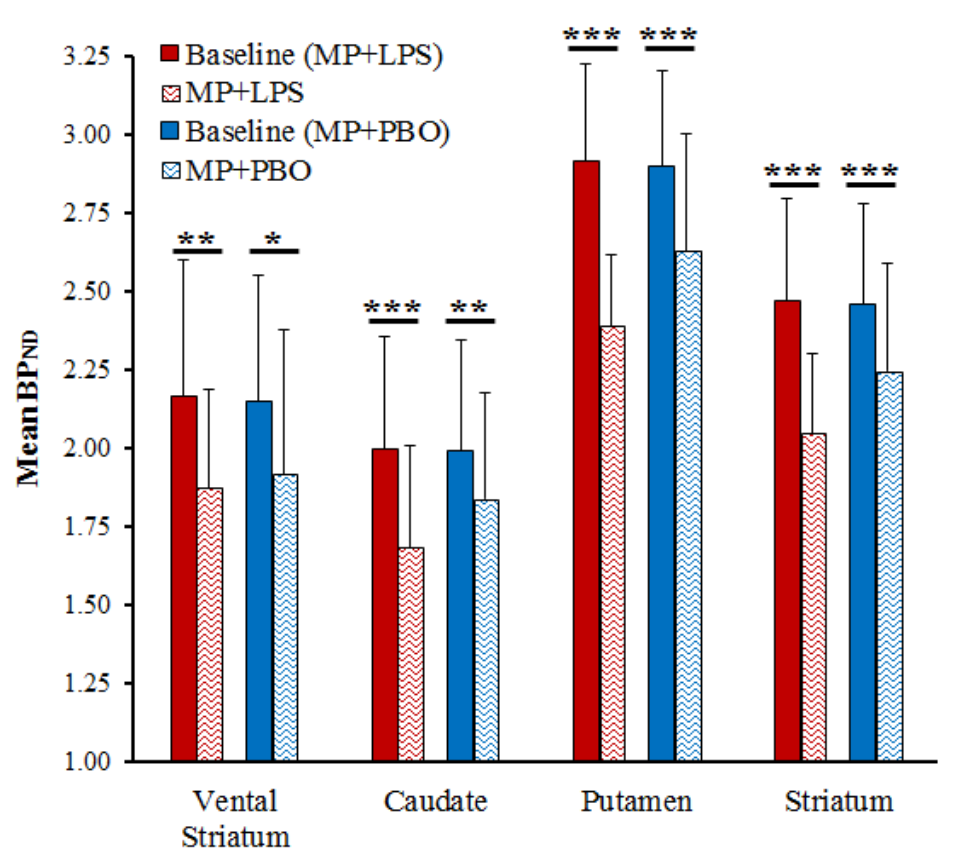

Supplement: Supplementary Figure [file tp201718x1.png]
