# Supplementary material for: Systemic inflammation enhances stimulant-induced striatal dopamine elevation
Source: Transl Psychiatry. 2017 Mar 28;7(3):e1076–. doi: 10.1038/tp.2017.18 (PMC5404612; doi:10.1038/tp.2017.18)
Supplement: Supplementary Figure Legend [file tp201718x2.docx]

**Supplemental Figure 1.** Mean BP across the 8 subjects in striatal ROIs, separated by scan type. Red bars indicate the MP+LPS condition and blue bars indicate the MP+PBO condition. Filled bars represent the mean BP in baseline scans while striped bars represent the mean BP in post-MP scans. The error bars represent standard deviation. Paired two-tailed t tests were performed between the baseline BPs and the corresponding post-MP BPs (*p<0.05, **p<0.005, ***p<0.0005) in each ROI.
